# Physiological Response, Oxidative Stress Assessment and Aquaporin Genes Expression of Cherry Tomato (*Solanum lycopersicum* L.) Exposed to Hyper-Harmonized Fullerene Water Complex

**DOI:** 10.3390/plants11212810

**Published:** 2022-10-22

**Authors:** Angelina Subotić, Slađana Jevremović, Snežana Milošević, Milana Trifunović-Momčilov, Marija Đurić, Đuro Koruga

**Affiliations:** 1 Department of Plant Physiology, Institute for Biological Research “Siniša Stanković”-National Institute of Republic of Serbia, University of Belgrade, Bulevar despota Stefana 142, 11060 Belgrade, Serbia; 2 TFT Nano Center, Vojislava Ilića 88, 11050 Belgrade, Serbia

**Keywords:** carbon nanosubstance, fullerene derivatives, fullerol, oxidative stress, lycopene, aquaporin gene

## Abstract

The rapid production and numerous applications of nanomaterials warrant the necessity and importance of examining nanoparticles in terms to their environmental and biological effects and implications. In this study, the effects of a water-soluble hyper-harmonized hydroxyl-modified fullerene (3HFWC) on cherry tomato seed germination, seedlings growth, physiological response and fruiting was evaluated. Changes in the photosynthetic pigments content, oxidative stress assessment, and aquaporin genes expression in cherry tomato plants were studied after during short- and long-term continuous exposure to 3HFWC nanosubstance (200 mg/L). Increased levels of photosynthetic pigments in leaves, lycopene in fruits, decreased levels of hydrogen peroxide content, activation of cellular antioxidant enzymes such as superoxide dismutase, catalase and peroxidase and increased aquaporin gene expression *(PIP1;3*, *PIP1;5* and *PIP2;4*) were observed in 3HFWC nanosubstance-exposed plants in comparison to control, untreated cherry tomato plants. The 3HFWC nanosubstance showed positive effects on cherry tomato seed germination, plantlet growth and lycopene content in fruits and may be considered as a promising nanofertilizer.

## 1. Introduction

Nanotechnology is a novel, innovative and multidisciplinary field of science that investigate the creation, manipulation and application of small materials and devices at molecular nanoscale (at least one dimension ranges in size from 1–100 nm). The benefits of nanotechnology are linked to advances in physics, chemistry, biology, medicine, pharmacology, environmental science, and agronomy [1]. Many studies have been devoted, over the last few decades, to the interaction of plants with nanoparticles and the development of new discipline in this field, phytonanotechnology [2]. Priorities in these research areas include the development of systems that allow controlled uptake, transport, and accumulation of nanomaterials, as well as their efficiency in the modulating of plant growth, development, and metabolism, as well as in reducing the effect of phytotoxicity [3]. Phytonanotecnology is a relatively new scientific field with enormous potential for the development of new tools in delivery of important agrochemicals, specific bioactive molecules required for manipulation in plant breeding, as well as genetic transformation [4].

**Figure 1:**
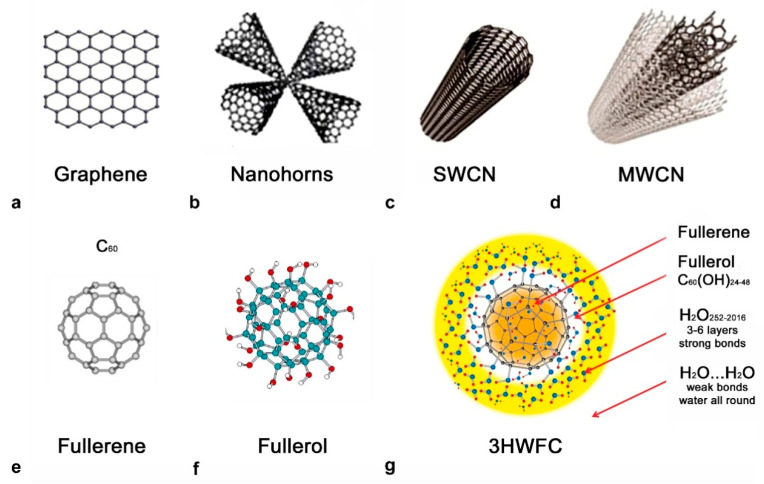
Different types of nanocarbon materials: (**a**) single sheeted graphene; (**b**) nanohorns; (**c**) single–walled nano tubes (SWCN); (**d**) multi–walled nanotubes (MWCN); (**e**) fullerene; (**f**) fullerol; and (**g**) hydroxylated fullerene water complex (3HFWC).

The application of nanomaterials in plant sciences and agronomy is constantly increasing, especially after discovering of carbon-based nanomaterials such as, sheeted graphene (Figure 1a), nanohorns (Figure 1b), single/multi-walled nanotubes (SWCNT/MWCNT, Figure 1c,d), nanodots and spherical fullerenes [2,3,5,6]. Fullerene (C_60_), the third allotropic modification of carbon besides graphite and diamond, was the first carbon-based nanomaterial discovered in 1985 [7]. The pristine form of fullerene consists of carbon atoms arranged in five and six-membered carbon rings, and the closed structure is provided by 12 pentagons (Figure 1e). Fullerene molecules may differ in size, i.e., the number of carbon atoms. The most famous is certainly the C_60_ molecule, whose structure is composed of 20 hexagons and 12 pentagons with the shape of a truncated polyhedron. This unique molecule, constructed of only one chemical element in the shape of a spherical cage, demonstrates the duality of wave and particle behavior, i.e., it has the properties of both an electromagnetic wave and a particle. It is widely used in various scientific disciplines due to its stability and ability to easily bind and release electrons, as well as its optical properties [8]. Unfortunately, pristine fullerene is practically insoluble in water (0.1 ng/L) and numerous investigations have been conducted to obtain functionalized, water-soluble derivatives and determine their effects on living systems [9]. The discovery of fullerols, C_60_(OH)_24_, a water-soluble molecule and the first fullerene derivative formed by symmetrical attachment of the 12–48 hydroxyl groups, solved this problem (Figure 1f). Hydroxylated fullerene (fullerols) showed strong free radical scavenging against reactive oxygen species (ROS) and antioxidative activity, demonstrating their multipotent biological usage [10,11]. Functionalized forms of fullerene have been recognized as powerful antioxidants with applications in chemotherapy, dermatology, neurodegenerative diseases and radiobiology that are very well established [12,13,14].

Tomato (*Solanum lycopersicum* L.) is an economically important plant species, that originated in South America and currently is common worldwide. The tomato plant is ranked as the fourth worldwide crop species besides coffee, banana, and rice [15]. It is cultivated because of its fruit, which has a much lower sugar content than other fruits. Furthermore, tomato fruit is classified as a protective food due to its high nutritive value which includes important nutrients such as carotenoids (lycopene, beta-carotene), flavonoids, vitamin C, and hydroxylcinamic acid derivatives [16]. It is consumed not only as a fresh food, but also as a processed food (juices, powder concentrates, soups and sauces). Tomato consumption and production have constantly increased over the last decade as a result of the discovery of lycopene’s anti-oxidative activity, antidiabetic, cardio-protective, anti-inflammatory, hepato- and neuro-protective, and anti-cancer effects [17,18,19], as summarized in [19]. Lycopene is a tetraterpene compound consisting of eight isoprene units and 11 double linear bonds. The color of tomato fruit is provided by this red pigment. Many studies have shown that lycopene is the most effective free radical scavenger among carotenoids. For instance, lycopene can remove singlet oxygen two to ten times more effectively than beta-carotene or tocopherol [20]. The cherry tomato is a smaller variety of tomato and has also been demonstrated to be beneficial to human health as it contains many antioxidants and secondary metabolites [21,22].

According to the available literature data, different nanocarbon materials have either positive, negative, or neutral effects on tomato growth, depending on nanoparticle type, concentration and growth conditions [summarized in Table 1]. All studies about the effects of nanocarbon derivatives on the tomato growth have included: (i) in vitro experiments with nanomaterials in nutrition media for seeds priming as described for SWCN [23,24,25,26,27], MWCN [24,28,29], nanohorns [30], graphene [31], graphene oxide [32] and fullerol [29]; (ii) foliar application as for MWCN [33]; or (iii) in hydroponic cultures as it is presented for MWCN [34], graphene [35] and graphene quantum dots [36]. There are few reports about the effects of nanocarbon materials on tomato growth when used as permanent nanofertilizers in soil. When SWCN and functionalized SWCN-OH were added to soil, tomato growth and flowering were delayed [37]. In contrast to SWCN, MWCN has no effect on the fresh biomass when it is supplemented in vermiculite [38]. When MWCN was added to the soil, however, contradictory results were obtained. In one report, increased plant height, flower and fruit formation of tomato plants were observed [39], while in another, tomato growth and flowering were delayed [37]. There is still a scarcity of data on the effect of fullerene or fullerols on tomato growth (Table 1). In general, fullerene and hydroxylated derivatives have a neutral effect on tomato growth [29,38,40]. Furthermore, when fullerene was added to vermiculite, no effect on growth, biomass and pesticide accumulation in tomato plants were observed [38,39].

Apart from tomato, a number of scientific studies during the last decade have described the various effects (positive and negative) of hydroxylated fullerenes on plant growth and development in other plant species. Investigations conducted on different plant species confirmed that fullerols may positively affect plant productivity under normal or stress conditions and could prevent the process of oxidative damage through radical scavenging activity [41,42,43]. Up to now, all investigations on the effect of fullerol derivatives on plant growth have included in vitro experiments with inclusion of fullerol derivatives directly in nutrition media; in seed priming for several hours or a couple of days; and in foliar and application in hydroponic systems. For instance, fullerol treatment of bitter melon seeds for 48h enhanced biomass, fruit yield, anticancer and antidiabetic secondary metabolite contents in bitter melon fruits [41]. Also, fullerol was stimulated hypocotyl elongation of *Arabidopsis thaliana* [44]. The foliar application of fullerol in drought-stressed *Beta vulgaris* alleviated oxidative stress [42]. Furthermore, the foliar application of fullerol improved salt tolerance in wheat through ion compartmentalization, osmotic adjustments and regulation of enzymatic antioxidants [45]. Fullerol improved seed germination, biomass accumulation, photosynthesis, and antioxidant activity in *Brassica napus* under water stress [46]. To the best of our knowledge, to date, there is only one in vitro study describing that fullerol and their derivatives had neutral effect on tomato seed germination and growth [29].

In this study, we used the second derivative of fullerenes, hyper-harmonized hydroxylated fullerene water complex (3HFWC, Figure 1g). This nanosubstance is made from C_60_(OH)_24–45_ fullerol, around which water layers are added. 3HFWC is a patented material created by the functionalizing of C_60_ molecule with OH groups (C_60_(OH)x), and through the addition of OH groups by the water layers (C_60_(OH)_36±12_@(H_2_O)_144–2528_. These water layers protect biomolecules from the potential toxic effects of C_60,_ as well as from the environmental influences. The entire nanosubstance is 6–18 nm in size and has no toxicity effects in the tests on human dermal fibroblasts and liver carcinoma cells [8,48]. It has a commercial application in cosmetics as an active component ingredient “hydroxylated fullerene” [13], and it has been shown to promote melanoma cell reprogramming toward a normal phenotype [49].

We investigated the influence of 3HFWC substance supplementation in soil on seed germination, growth, fruiting, and lycopene content in cherry tomato fruits. The morphological and physiological responses, oxidative stress assessment and aquaporin gene expression were compared among cherry tomato plants continuously exposed to 3HFWC substance and control, untreated plants.

## 2. Results

### 2.1. The Effect of 3HFWC Nanosubstance on Cherry Tomato Seed Germination, Seedlings Growth and Development

**Figure 2:**
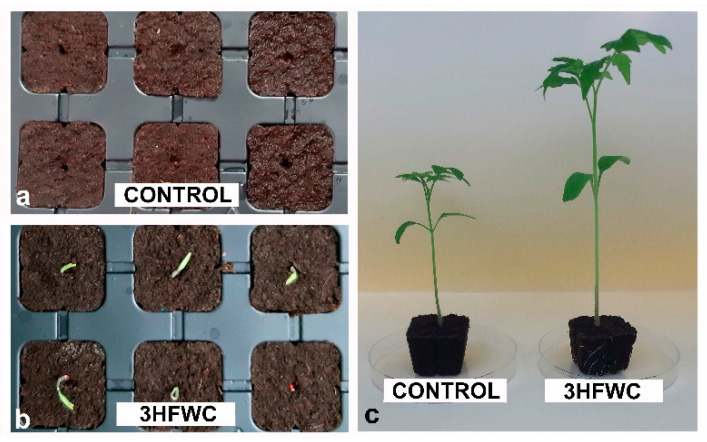
The effect of 3HFWC nanosubstance on cherry tomato seed germination: (**a**,**b**) growth containers with control seeds; (**a**) 3HFWC nanosubstance-treated seeds; (**b**) after three days of growth; and (**c**) cherry tomato seedlings after three weeks of growth.

The growth-promoting effect of 3HFWC nanosubstance (200 mg/L) on cherry tomato seeds was obvious within the first days of cultivation. Even after three days of cultivation, 3HFWC nanosubstance-treated seeds showed early seedling development when compared to control seeds (Figure 2a,b). After 10 days of developments, control seeds germinated at the same rate as for 3HWFC nanosubstance-exposed seeds. After three weeks of growth, the germination rate reached 98% in both experimental groups, with no statistically significant difference between control (control plants) and 3HFWC nanosubstance-treated seedlings (3HFWC plants). However, the growth parameters of developed seedlings varied greatly between these experimental groups of plants (Figure 2c, Table 1).

The 3HFWC seedlings showed significantly better growth than the control once (Table 2). 3HFWC-exposed seedlings were 41.7% higher than control seedlings. The fresh weight of 3HFWC seedlings was 3.4 times higher than of the control, and more leaves were developed on each seedling (Table 2).

### 2.2. The Effects of Short and Long-Term Exposure to 3HFWC Nanosubstance on Cherry Tomato Physiological and Molecular Response

Photosynthetic pigments content and oxidative stress assessment in plants were evaluated after four weeks (short-term) and 12 weeks (long-term) of continuous exposure to 3HFWC nanosubstance.

#### 2.2.1. Photosynthetic Pigments Content

**Figure 3:**
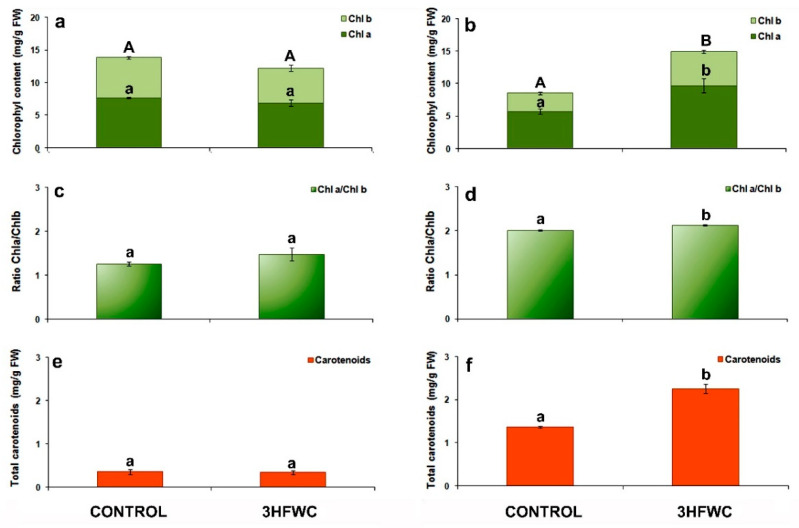
The effect of 3HFWC substance on pigment content in cherry tomato leaves. (**a**,**b**) Chlorophyll a (*Chl a*) and chlorophyll b (*Chl b*) content in cherry tomato leaves after 4 (**a**) and 12 weeks (**b**) of growth; (**c**,**d**) *Chl a*/*Chl b* ratio in cherry tomato leaves after 4 (**c**) and 12 weeks (**d**) of growth; (**e**,**f**) Total carotenoid content in cherry tomato leaves after 4 (**e**) and 12 weeks (**f**) of growth. The presented data are mean value ± standard error. Different letters indicate statistically significant differences according to LSD test (*p* ≤ 0.05).

Chlorophyll a and b (*Chl a*, *Chl b*) content, their ratio and total carotenoid content of cherry tomato (Figure 3a–f) evaluated after short-term exposure are presented in Figure 3a,c,e and after long-term exposure in Figure 3b,d,f. After short-term exposure there was no differences in photosynthetic pigment content between cherry tomato plants treated with 3HFWC nanosubstance and control plants (Figure 3a,c,e). After long-term exposure, cherry tomato plants showed a significant increase in photosynthetic pigments content (Figure 3b,d,e).

The total chlorophyll content *Chl a* + *Chl b* (67.36%) and carotenoid content (64.9%) in 3HFWC nanosubstance-treated plants were higher than in control tomato cherry plants. A higher total *Chl* content in 3HFWC nanosubstance-treated plants resulted from the higher levels of both chlorophylls, *Chl a* (70.3%) and *Chl b* (84.7%). Also, the *Chl a*/*Chl b* ratio in 3HFWC nanosubstance-exposed plants was 5.9% higher than in control tomato cherry plants.

#### 2.2.2. Oxidative Stress Assessment

**Figure 4:**
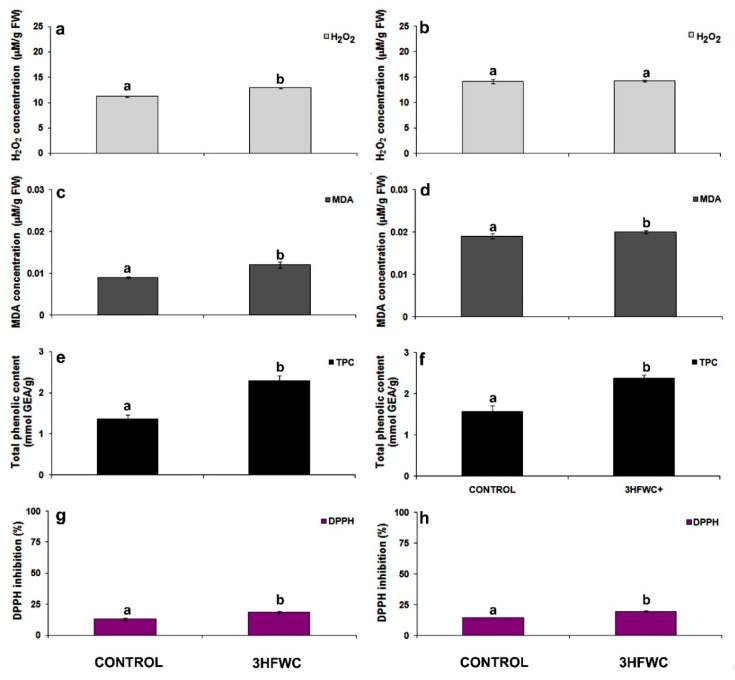
The effect of 3HFWC substance on hydrogen peroxide (H_2_O_2_), malondialdehyde (MDA), total phenolic content (TPC) and DPPH activity in cherry tomato leaves: (**a**,**b**) H_2_O_2_ content in cherry tomato leaves after four (**a**) and 12 weeks (**b**) of growth; (**c**,**d**) MDA content in cherry tomato leaves after four (**c**) and 12 weeks (**d**) of growth; (**e**,**f**) TTC content in cherry tomato leaves after four (**e**) and 12 weeks (**f**) of growth; (**g**,**h**) DPPH activity cherry tomato leaves after four (**g**) and 12 weeks (**h**) of growth. The presented data are mean value ± standard error. Different letters indicate statistically significant differences according to LSD test (*p* ≤ 0.05).

After short-term exposure to 3HFWC nanosubstance, hydrogen peroxide (H_2_O_2_) content was significantly increased in tomato cherry plants (Figure 4a). The H_2_O_2_ content in plants was over than 15.2% higher in 3HFWC growing plants than in control ones. After long-term exposure, there were no statistically significant differences in H_2_O_2_ content between 3HFWC nanosubstances treated and control plants (Figure 4b). Contrary to H_2_O_2_, malondialdehyde (MDA) content in 3HFWC nanosubstance-treated plants was increased during the whole period of cultivation. Significant increment of MDA content after short-term cultivation (33.3%) was observed. MDA increment in 3HFWC nanosubstance-treated plants after long-term cultivation was less (5.3%) than after short-term cultivation.

**Figure 5:**
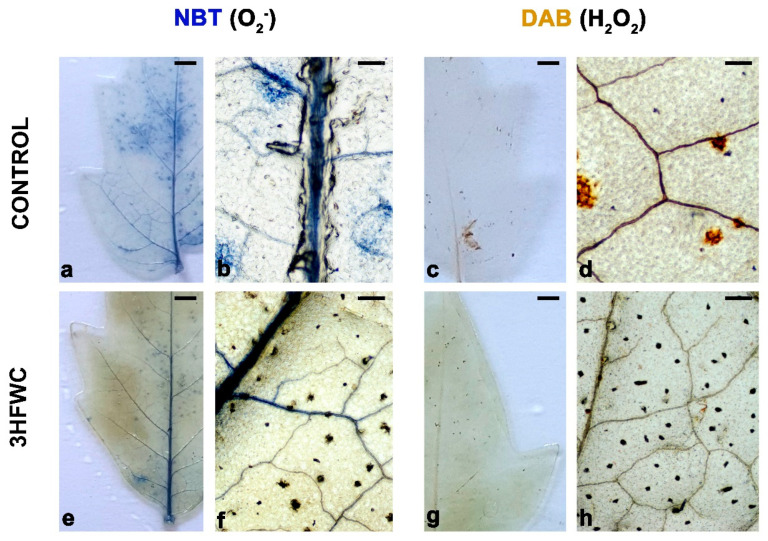
Localization of O_2_^−^ (NBT staining) and H_2_O_2_ (DAB staining) in the leaves of control and 3HFWC substance treated cherry tomato plants after 12 weeks of growth: (**a**,**b**) localization of superoxide anion O_2_^−^ in whole leaf (**a**) and leaf midrib and veins of control plants (**b**); (**c**,**d**) accumulation of H_2_O_2_ in whole leaf (**c**) and leaf midrib and veins of control plants (**d**); (**e**,**f**) localization of superoxide anion O_2_^−^ in whole leaf (**e**) and leaf midrib and veins of 3HFWC substance treated plants (**f**); and (**g**,**h**) accumulation of H_2_O_2_ in whole leaf (**g**) and leaf midrib and veins of 3HFWC substance treated plants (**h**). Bars: **a**,**c**,**e**,**g** 5 mm; **b**,**d**,**f**,**h** 200 μm.

After long-term exposure, we investigated the localization of ROS production (superoxide anion O_2_^−^ and H_2_O_2_) in leaves of 3HFWC nanosubstance-exposed and control plants (Figure 5). At first glance, it was obvious that total bleaching was possible only on control plants (Figure 5a–d) due to the significantly higher pigment content in 3HFWC nanosubstance-treated plants (Figure 3b,f and Figure 5e,h). The production of superoxide anion O_2_^−^ (Figure 5a,b,e,f) and accumulation of H_2_O_2_ (Figure 5c,d,g,h) were localized mainly in the leaf midrib and veins. In the leaves of 3HFWC-treated plants the deposits of 3HFWC nanosubstance were also noticed (Figure 5f,h).

The total phenolic content (TPC) increased consistently after the exposure to 3HFWC nanosubstance (Figure 4e,f). In comparison to the control plants, TPC increased by 67.1% (Figure 4e) after short- term exposure to 3HFWC nanosubstance. TPC content was higher (by 50.6%) in long-term exposed 3HFWC plants than control plants (Figure 4f).

In general, we observed significantly higher antioxidant capacity of leaves, determined by DPPH radical (1,1′-diphenyl-2-picrylhydrazyl), in 3HFWC nanosubstance grown than in control plants (Figure 4g,h). In comparison to the control plants, antioxidant capacity after short-term exposure was higher in 3HFWC nanosubstance grown plants, by 42.0% (Figure 4g), but also after long-term exposure by 50.6% (Figure 4h).

#### 2.2.3. Antioxidative Enzyme Activity

**Figure 6:**
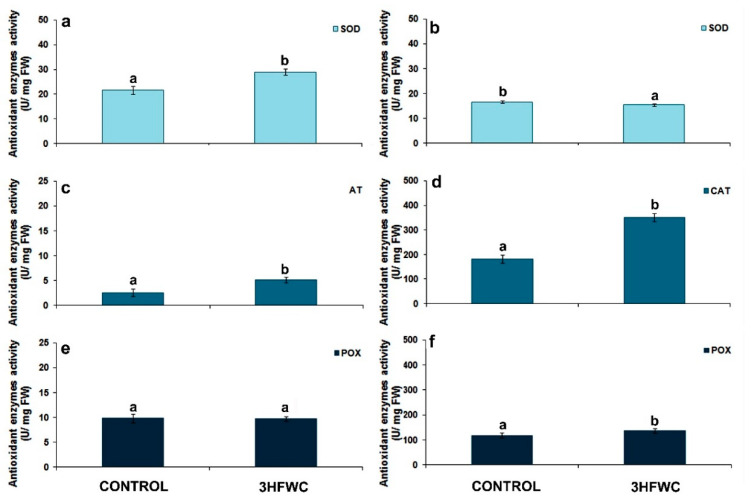
The effect of 3HFWC nanosubstance on superoxide dismutase (SOD), catalase (CAT) and peroxidase (POX) activity in cherry tomato leaves: (**a**,**b**) SOD activity in cherry tomato leaves after short- (**a**) and long-term growth(**b**); (**c**,**d**) CAT activity in cherry tomato leaves after four (**c**) and 12 weeks (**d**) of growth; (**e**,**f**) POX activity in cherry tomato leaves after four (**e**) and 12 weeks (**f**) of growth. The presented data are mean value ± standard error. Different letters indicate statistically significant differences according to LSD test (*p* ≤ 0.05).

The activity of antioxidative enzymes superoxide dismutase (SOD), catalase (CAT) and peroxidase (POX), were evaluated after short-term (Figure 6a,c,e) and long-term exposure to 3HFWC nanosubstance (Figure 6b,d,f). Thought the whole period of cultivation, SOD activity in cherry tomato plants ranged 15.4–28.9 U/mg FW. SOD activity was increased by 30.37% in plants exposed to 3HFWC nanosubstance after short-term treatment (Figure 6a) but decreased by 18.9% after long-term cultivation. Contrary to SOD, the activity of other two antioxidative enzymes varied significantly during short-term and long-term cultivation. Generally, the CAT activity was seventy times higher, while POX activity was twelve times higher after long-term growth of cherry tomato than after short-term growth.

Short term exposure to 3HFWC nanosubstance significantly increased the CAT activity (Figure 6c) by 100% when compared to control plants. This trend remained during long-term cultivation, with a 98.3% increase in CAT activity (Figure 6d). In contrast to SOD and CAT, there was no difference in POX enzyme activity between control and 3HFWC nanosubstance-exposed tomato cherry plants during short-term cultivation (Figure 5e). After long-term cultivation, the activity of this enzyme was higher in 3HFWC nanosubstance-grown plants by 18.9% than in control plants.

### 2.3. Histological Analysis of Cherry Tomato Plants after Long-Term Exposure to 3HFWC Nanosubstance

**Figure 7:**
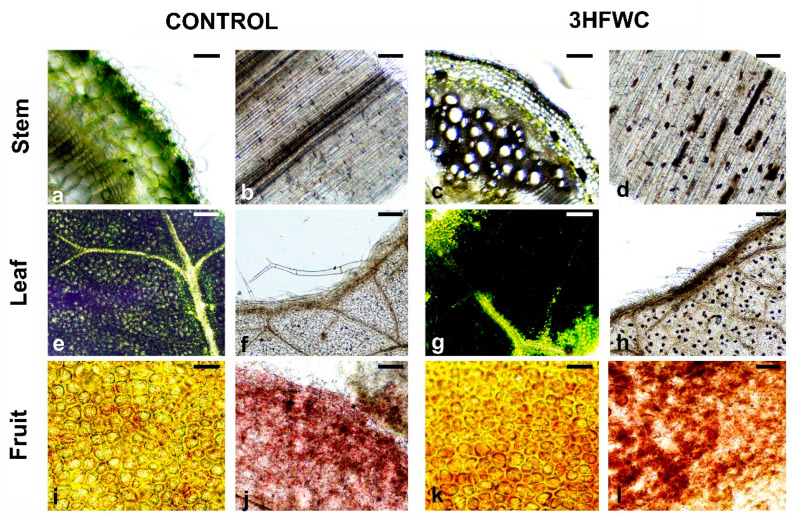
Histological analysis of control and 3HFWC grown plants after 12 weeks of growth. (**a**–**d**) Cross sections of stems of control (**a**,**b**) and 3HFWC-exposed plants (**c**,**d**), transversal sections (**a**,**c**), longitudinal sections (**b**,**d**); (**e**–**h**) Leaves of control (**e**,**f**) and 3HFWC (**g**,**h**) exposed plants, fresh leaf (**e**,**g**), bleached leaf (**f**,**h**); (**i**–**l**) Fruits of control (**i**,**j**) and 3HFWC-exposed plants (**k**,**l**), peal (**i**,**k**), pulp (**j**,**l**), Bars, 200 μm.

Detailed histological analysis revealed that 3HFWC nanosubstance deposits accumulated in the tomato vascular elements of the stem and leaf lamina after 12 weeks of growth (Figure 7). In the stem, discrete deposits of accumulated 3HFWC nanosubstance were observed as randomly spherical or conical deposits along xylem vascular elements (Figure 7a–d). Spherical deposits were observed in the leaves of plants exposed to 3HFWC nanosubstance, mainly in leaf lamina (Figure 7e,h). In contrast to stem and leaves, the deposits or any visible accumulation of 3HFWC nanosubstance in cherry tomato fruits were not observed (Figure 7i,j).

### 2.4. Fruit Quality of 3HFWC Nanosubstance Exposed Cherry Tomato Plants

**Figure 8:**
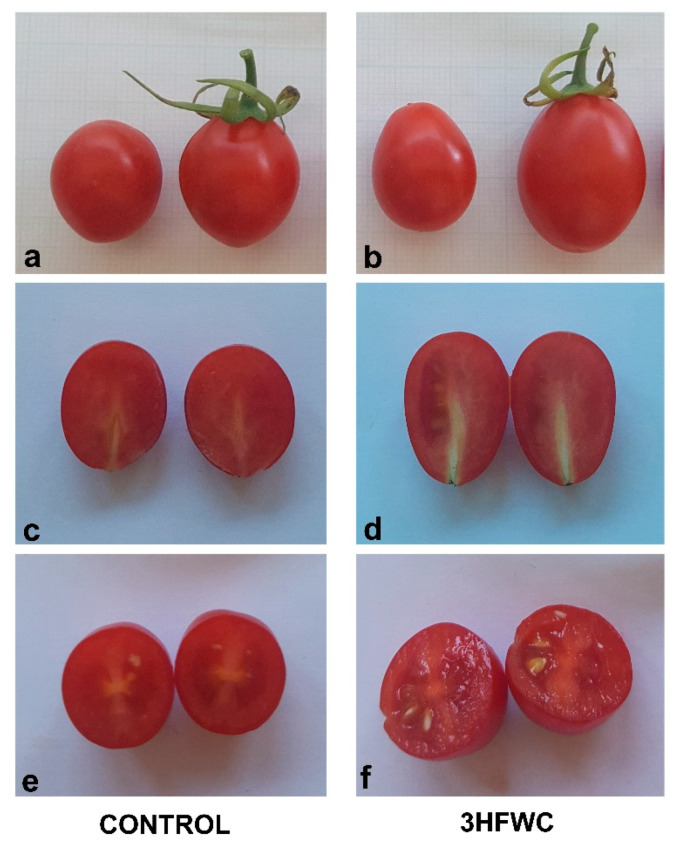
The morphological analysis of cherry tomato fruits derived after exposure to 3HFWC nanosubstance. (**a**,**b**) Cherry tomato fruits of control (**a**) and 3HFWC-exposed plants (**b**); (**c**,**d**) Longitudinal section of fruits of control (**c**) and 3HFWC-exposed plants (**d**); (**e**,**f**) Transversal section of control (**e**) and 3HFWC nanosubstance-exposed plants (**f**).

Fruits derived from 3HFWC nanosubstance-exposed cherry tomato plants significantly differed compared to control plants (Table 3, Figure 8). Fruits were 32.5% heavier than the control once (Table 3). In addition, the fruits of 3HF WC nanosubstance-exposed cherry tomato plants (Figure 8b,d,f) were wider (by 8.7%) and longer (by 12.9%) than the fruits of control plants (Figure 8a,c,e).

**Figure 9:**
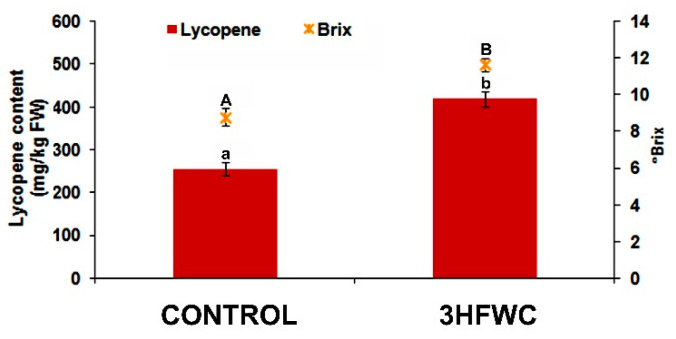
The lycopene content and BRIX° in fruits of control and 3HFWC nanosubstance-exposed cherry tomato plants. The presented data are mean value ± standard error. Different letters indicate statistically significant differences according to LSD test (*p* ≤ 0.05).

The most significant difference between fruits derived from 3HFWC nanosubstance-exposed cherry tomato plants was the content of lycopene (Figure 9).

The lycopene content in fruits from 3HFWC nanosubstance grown plants was 63.8% higher than in fruits from control plants. Furthermore, the BRIX° of fruits from 3HFWC nanosubstance-exposed plants was 32.9% higher than in the fruits of control plants (Figure 9).

### 2.5. The Effect of 3HFWC Nanosubstance Onaqaporine Gene Expresion

**Figure 10:**
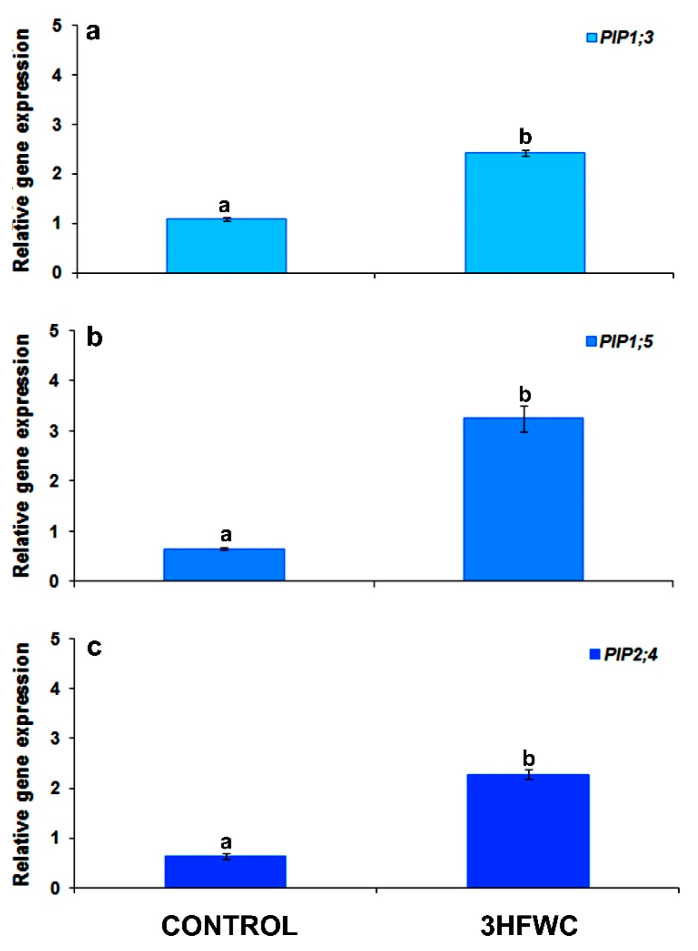
The effect of 3HFWC nanosubstance on relative expression of aquaporin genes in cherry tomato leaves after long-term exposure: (**a**–**c**) relative gene expression of *PIP1;3* (**a**); *PIP1;5* (**b**); and *PIP2;4* (**c**). The gene expression was analyzed by the qRT-PCR method and normalized to the expression to 18s RNA. Data represent mean values ± standard error of three technical replicates. Different letters indicate statistically significant differences according to Duncan’s test (*p* ≤ 0.05).

All three investigated aquaporin genes (*PIP1;3*, *PIP1;5*, *PIP2;4*) had higher relative expression in 3HFWC nanosubstance-exposed plants than in the control plants (Figure 10). Relative expression of *PIP1;3* gene was 220% higher (Figure 10a), while expression of *PIP2;4* was 360% higher (Figure 10c) in plants exposed to 3HFWC nanosubstance than in control plants. The highest difference in relative expression was observed for the *PIP1;5* gene, which expression was 500% higher in 3HFWC nanosubstance-exposed plants in comparison to control plants (Figure 10b).

## 3. Discussion

Wide developments in the field of nanotechnology have enabled the use of carbon-based nanomaterials in numerous and different areas of plant system growth and regeneration [50]. Because of their water solubility, newly discovered functionalized fullerenes have a wide range of potential applications. There are many data about the effects of different types of fullerenes on plant growth but the results of effects of hydroxylated fullerene derivatives (fullerols) are sporadic and still scarce, as summarized in [51]. We presented, for the first time, the results about the application of a new generation of nanosubstance, hydroxylated fullerene derivative (3HFWC nanosubstance), and evaluated their effect on plant growth. In our research, we used cherry tomato as an experimental model plant, because it reaches maturity relatively quickly (70–90 days) and can complete the entire process until fruiting under greenhouse conditions (GrowBoxes). We revealed that 3HFWC made significant improvements in cherry tomato seed germination, plant growth, photosynthetic pigments content and antioxidant ability due to increased water and nutrient uptake.

To date, all investigations about the effect of hydroxylated fullerene derivatives on plant growth have included: (i) in vitro experiments, such as one used for onion [52] and *A. thaliana* [44]; (ii) foliar application in sugar beet [42], (iii) seeds priming of bitter melon [41], tomato [29], corn ([53], barley [54] and wheat [45,55] or in hydroponic cultures of wheat [2,43] and cucumber [56]. Studies on the effects of fullerol and its derivatives on the plant development and growth when they are supplemented continuously in soil as nanofertilizers are still lacking. Accordingly, this study represents a significant contribution to knowledge about the effects of one specific fullerene derivative on plant development and growth. According to our results, the 3HFWC nanosubstance had positive effects on seed germination and the overall plant growth of cherry tomato plants. It can be concluded that 3HFWC nanosubstance is safe for use and did not show any negative effects on cherry tomato growth, so we believe it could be considered for use as a potential nanofertilizer.

### 3.1. The Effect of 3HFWC Nanosubstance on Cherry Tomato Seed Germination and Seedling Growth

Exposure to 3HFWC nanosubstance significantly improved seed germination of cherry tomato. Generally, plant responses to carbon-nanomaterials vary depending on the type, the functional group present on nanomaterials as well as plant species. Seed germination is the first step and the most sensitive growth phase of higher plants. Carbon nanomaterials have been shown to have positive, neutral, or negative effects on seed germination in various plant species [50]. The same effects of different nanomaterials are also reported for tomato [23,28,37]. Multi-walled nanotubes showed a positive effect on tomato seed germination, they penetrated the coats of seeds and germination began with high imbibition and activation of water channels [28,29]. Single-walled nanotubes, on the other hand, inhibited root elongation [23], and delayed ex vitro shoot growth [26,37]. Besides these, hydroxylated fullerene derivatives (fullerol) also showed a neutral effect on the germination percentage and length of tomato seedlings [29]. Priming wheat seeds for seven days with fullerol resulted in root elongation while biomass gain of the leaves and stems remain unaffected [57]. Limited studies have considered the role of fullerene or their derivatives during direct growing of tomato seeds in soil substrates (vermiculite) [38,40].

Tomato plant biomass and pesticide accumulation were unaffected by a 20-day expo-sure to fullerene supplemented with vermiculite [38,40]. In our study, we revealed strong promotion of cherry tomato seed germination followed by stimulated seedlings growth after expose to 3HFWC nanosubstance. The promotive effect of 3HFWC nanosubstance on cherry tomato seed germination was obvious even after a few days after sowing in soil.

The root is a prime organ for absorbing water and nutrients through the soil. The main sites for water uptake are permeable root tips and hairs, while exodermis is hydrophobic. Nano carbon materials have shown to improve water conductivity in plants [39,41]. It is postulated that water-soluble nanocarbon substances enter from the soil via symplastic transmembrane pathways (xylem vessels) and are then translocated into the aerial part of plants [58,59]. Nanocarbons in xylem vessels can facilitate water flow which could be directly related to increased nutrient flow. From the root cortex, nanocarbons are further, through the xylem, transported in various parts of plants like leaf, stem, fruit, and flower. The reason for increased germination of 3HFWC nanosubstance-exposed cherry tomato seeds lies in the insolubility of these fullerene derivatives in water, and their ease of movement within the plant cell. As a result, increased water uptake necessary for further seed imbibition and better seed germination was achieved. The toxic nature of un-functionalized nanocarbon materials is thought to be a physical phenomenon caused by their sufficient stiffness to rupture the soft membrane of a cell [59]. In contrast, functionalized nanocarbon substances, such as hydroxylated fullerenes, are highly dispersed and can move readily inside the cell. The high degree of nanocarbon substance surface functionality remarkably reduces the hardness and toxicity effects [23,28,59,60]. 3HFWC nanosubstance has functionalization of C_60_ molecule with OH groups (C_60_(OH)x) and through the addition of OH groups by water layers C_60_(OH)_36±12_@(H_2_O)_144–2528_. These water layers surround the solid phase—hydrogen bonded C_60_(OH)_36±12_ nanostructure, and protect complex from the environmental influences, and at the same time, they protect biomolecules from the potential toxic effects of the C_60_. The improved water status in cherry tomato seeds exposed to 3HFWC nanosubstance increased seed germination and overall better seedlings and plantlet growth. Plantlets exposed to 3HFWC nanosubstance successfully grown during the whole vegetative growth, flowered, and produced fruits. It is evident that the structure of 3HFWC nanosubstance, whose diameter size ranges between 8–12 nm had no toxicity effects on cherry tomato as it has already shown for human cells [13,48]. The bio-distribution of 3HFWC nanosubstance in cherry tomato plants can be visualized as numerous black aggregates (deposits) in bleached plant tissues (xylem and leaves) after prolonged exposure. In a study with bitter melon the fullerol C_60_(OH)_20_ deposits were also detected in different plant parts [41]. On the other hand, fullerol deposits were mainly distributed in roots with minor amounts in the stems and leaves of wheat [57].

### 3.2. The Effect of 3HFWC Nanosubstance on Photosynthetic Pigment Content

The content of photosynthetic pigments cherry tomato leaves increased significantly when exposed to the 3HFWC nanosubstance. Previous studies reported that fullerene derivatives can stimulate photosynthetic pigments and growth characteristics of plants [43,57]. Increased water uptake in plants positively correlates with increased uptake of beneficiary nutrients, which resulted in improved assimilation and photosynthetic capacity. It is well documented that fullerol promoted *Chl* synthesis in wheat throughout a one week-long examination period [57]. Also, long-term exposure (20 weeks) to MWCN of barley, soybean, and corn, showed no significant toxic effects on the plant development. Furthermore, the enhancement of photosynthesis in MWCNTexposed crops was also observed [61]. The results obtained in our study are in concordance with these findings. Long-term exposure to 3HFWC nanosubstance resulted in a higher content of photosynthetic pigments (both *Chl* and carotenoids) which contributed significantly to improved cherry tomato growth, probably by enhancing photosynthesis efficiency.

### 3.3. The Effect 3HFWC Nanosubstance on ROS Production and Antioxidant Properties

In nature, plants grow in a variety of agricultural environments and faced multiple abiotic stresses which limit their productivity. All environmental stress factors cause overproduction of ROS within cellular/sub-cellular compartments. Considering that oxidative stress can disturb and damage vital biomolecules, the balance between ROS production and neutralization is very important in plants exposed to different abiotic and biotic stresses. There are numerous reports about various engineered nanomaterials as potential candidates for improving plant nutrient status, but, at the same time, there are few reports on the use of nanomaterials to improve oxidative stress tolerance and enhance antioxidant capacity in plants [46,51]. This study could help us to understand how complex biological systems, such as whole plants, respond to long-term exposure to hydroxylated fullerene derivatives. According to the findings presented in this work, 3HFWC nanosubstance not only enhanced plant growth, but also changed and regulated ROS production as well as elevated the antioxidant properties of cherry tomato plants.

There is some evidence that hydroxylated fullerene derivatives could enhance plant growth due to their ability to regulate oxidative stress [42,45,54]. It is postulated that hydroxylated fullerene act as molecular antioxidants and have a high ROS scavenging capacity. These nanocarbon substances have been shown to have an antioxidant effect in animals, where they neutralize multiple ROS forms and mimic the action of naturally occurring antioxidant molecules and enzymes in the cell [60,62]. These results were based on the fact that fullerols behave as radical sponges with the ability to neutralize almost all ROS on one side [45,63,64,65] and stimulate the antioxidant system to scavenge ROS by activation of antioxidant enzymes on the other side [42,53]. In our study long-term exposure to 3HFWC nanosubstance induced an oxidative defense response, enhanced antioxidant capacity and equilibrate H_2_O_2_production in cherry tomato plants.

In general, it has been shown that fullerol has SOD properties in plants, suggesting that it may clear ROS directly [51,66,67]. The proposed direct reaction of fullerols with superoxide anion consider the action of fullerol as a catalyst which uses two molecules of O_2_^−^ and water, resulting in H_2_O_2_, molecular oxygen, and a couple of hydroxyl radicals, and on that way mimic SOD activity [65,68]. SOD is the first line of defense against ROS toxicity capable of converting O_2_^−^ to H_2_O_2_ [69]. In cherry tomato exposed to 3HFWC nanosubstance cellular SOD enzyme activity was lower than in control plants after long-term exposure. We can postulate that 3HFWC nanosubstance assumed some of SOD radical scavenging function in cells. In addition, the fullerol-mediated scavenging of other ROS (i.e., OH^−^ radicals) can be explained in two possible routes. The first route involves that the addition of an OH^−^ radical is possible to the abundant sp2 carbon atoms in fullerol molecules. Secondly, the free radicals (i.e., OH^−^) tend to abstract e- of hydrogen from the fullerol-core, resulting in a stable polyhydroxy fullerene radical [67,70,71]. The radical elimination by lower fullerols (with fewer OH groups) can be linked with sp2 carbons and OH^−^ radical elimination by an addition reaction. In contrast, the fullerols with more OH groups, such as 3HFWC nanosubstance, tend to eliminate OH^−^ via the second H-abstraction mechanism [51,65]. The efficiency of 3HFWC nanosubstance as a radical sponge, like that of all hydroxylated fullerenes, lies in a fact that a single molecule can quench multiple types of free radicals. As a result, they are much more beneficial than naturally occurring antioxidants which generally act as specific radical quenchers and can only handle one or two electrons at a time [51]. Our results confirmed the ability of a new generation of hydroxylated fullerenes to modulate oxidative stress response and H_2_O_2_ production as well as activation of stress-related enzymes.

### 3.4. The Effect of 3HFWC Nanosubstance on Aquaporin Genes Expression

In general, plasma membrane aquaporins’ gene expression was elevated by exposure to 3HFWC nanosubstance of cherry tomato. Aquaporins are transmembrane proteins that serve primarily as channels for water and other solutes transport of across the plant membranes [72]. They can be found in all plant organs, such as roots, stems, leaves, flowers, fruits and seeds. Most of them were discovered in plasma membranes and tonoplast, but their presence is also reported in other intracellular membranes. The largest plant aquaporin subfamily, Plasma membrane Intrinsic Proteins (PIPs), is divided into two subgroups, PIP1 and PIP2, with distinct localization and functions in water and solute transport across plant membranes [72]. Members of the PIP1 and PIP2 subgroups can also transport other molecules such as CO_2_, glycerol, H_2_O_2_ and boron through membranes [73,74,75]. PIP2 members are efficient water channels and recent evidence indicates that PIP1 proteins require heterotetramerization with PIP2 to participate in water transport [74,76,77]. It is very well documented that some nanocarbon substances (MWCN) may facilitate water conduction and increase aquaporin’s gene expression in tomato seeds, roots, or individual plant cells [24,28,39]. In our work, we recorded significant activation of gene expression of all investigated aquaporin proteins isoforms after exposure to 3HFWC nanosubstance. Increased expression of these genes improved water and solute conduction throughout the whole plant which significantly contributed to improved cherry tomato growth. Although the influence of nanosubstances cannot be explained through changes in the expression pattern of a few genes for plasma membrane aquaporin proteins, further investigation is necessary to elucidate the mechanisms by which certain nanosubstances can induce the expression of specific genes.

### 3.5. Effect of 3HFWC Nanosubstance on Fruit Size and Lycopene Production

Exposure to 3HFWC increased the fruit size and lycopene content in cherry tomato fruits. Apart from physiological and phenotypic responses, the effect of nanocarbon substancies on useful secondary plant metabolotes is still sporadic. The concept of “nano-elicitors” has recently emerged as a novel approach to stimulate the production of valuable compounds [78] that could be used as pharmaceutical products and additives in food and cosmetics [79]. For instance, the accumulation of fullerol resulted in increase in biomass and fruit yield, as well as valuable secondary metabolites content in bitter melon fruits [41]. Priming of bitter melon seeds for 48h with fullerol resulted in higher amounts of inulin (up to 91%), charantin (up to 20%), lycopene (up to 82%), and cucurbitacin-B (up to 74%) in fruits, compared to fruits of non-primed seeds [41]. Tomato plants grown on soil supplemented with nanotubes (MWCN), produced the same number of leaves but twice as many flowers and fruits than plants grown in regular soil [39]. Also, the number of seeds and size of fruits per plant were not affected by application MWCN of tomato plants. Our results indicated that exposure to 3HFWC nanosubstance significantly increased the fruit size, lycopene content and BRIX in cherry tomato fruits. These results indicate that 3HFWC nanosubstance can be efficient for the activation of valuable secondary metabolites in tomato fruits.

The impact of tomato fruits containing absorbed nanosubstance on animal and human health is a critical step in risk assessment for a new phytonanobiotechnology field. To our knowledge, there is only one report about the effects of nanotubes (MWCN) containing tomato fruits on the human intestinal microbiota and gastrointestinal epithelial cell barrier function [80]. According to described results there were no major toxic effect of extracts from these tomato fruits at the cellular or gene expression levels of human epithelial cells [80]. On the other hand, there is wide range of fullerene-based skincare products that have been clinically verified and used by people for several years, implying that fullerenes and related materials are quite safe for topical application [81]. Compared to the control groups, cosmetic products containing 3HFWC nanosubstance demonstrated positive effects, including faster collagen regeneration and prompt skin reaction to the negative environmental influences [13,82]. The in vitro studies with melanoma cells recently revealed that 3HFWC nanosubstance treatments induced cell reprogramming, suggesting their use as a nonaggressive strategy for tumor growth suppression [49]. Despite these promising results, future in vitro and in vivo experiments with 3HFWC substance-containing fruits, could help in elucidating the influence of these nanosubstance on human health.

## 4. Materials and Methods

### 4.1. Plant Material, 3HFWC Nanosubstance Treatment and Growth Conditions

All experiments were conducted with an indeterminate hybrid of cherry tomato (*Solanum lycopersicum* L.) cv. “Read Heart 1” (YÜSKEL TOHU, Aksu, Antalya,Turkey). Seeds (24) of the selected cultivar were sown in nutrient cubes of coconut substrate (EazyPlug 24, EasyPlug, Goirle, Netherlands) and grown in greenhouse inside of three GrowBoxes 150 × 150 × 200 cm (Hidroponica, Belgrade, Serbia) which enable control of photoperiod as well as temperature and humidity during cherry tomato growth. In each GrowBox, in parallel to control (untreated) seeds, the effect of 3HFWC nanosubstance (200 mg/L) on cherry tomato seed germination and growth till fruiting was evaluated. During the germination phase, control seeds were irrigated with water, while treated seeds were irrigated with 3HFWC nanosubstance once a day at a two-day interval. When seedlings developed the first leaves, plants were transplanted into 10 × 10 cm pots filled with the “Plagron Light Mix” substrate (Plagron, Ospel, Netherlands). The substrate is slightly enriched, composed of a mixture of selected types of peat (black peat 55.6%, white peat 37%), perlite 7.5%, and peat enriched with plant growth fertilizer mix NPK 12:14:24 (1.5 kg/m^2^). “Hesi Soil Grow schedule” feeding program was implemented after transplanting the seedlings in pots ones a week. During the vegetation, the standard agrotechnics for cherry tomato grow indoors under the conditions of controlled irrigation was applied. When the plants reached a height of 7 to 15 cm, they were transplanted into “AutoPot Easy2Go” systems (Auto Pot Ltd. Hampshire, UK) for further cultivation in GrowBOXes until full physiological maturity and fruiting (12 plants per treatment). During cultivation in “AutoPot Easy2Go” systems, the same feeding program was implemented as for control plants; once a week but with addition of 3HFWC nanosubstance (200 mg/L).

The GrowBoxes’ photoperiod was 18h light/6 h dark with 280 µmol m^−2^ s^−1^ (photosynthetic photon flux density, PPFD) irradiance by daylight led lamps. The natural light intensity at the top of the canopy was estimated to be at least 300 µmol m^−2^ s^−1^ at 12 a.m. Temperature in BOXs varied 25 ± 2 °C, during the daylight regime and 18 ± 2 °C during the night. The relative humidity was set at 60 ± 5% during the day and maintained at 80 ± 5% during the night.

### 4.2. Measurement of Morphological Parameters of Seedlings and Fruits

Cherry tomato seedlings height, fresh weight and number of developed leaves were evaluated after three weeks of control and 3HFWC nanosubstance-treated seedlings. For fruits, the measurements regarding fresh weight, equatorial and longitudinal diameters of control and 3HFWC nanosubstance grown plants were performed during the fruiting phase. All measurements of diameters of each tomato were measured using a digital caliper (model 500-197-20 150 mm; Mitutoyo Corp., Aurora, IL, USA).

### 4.3. Histological Analysis

The pieces of fresh plant material (stem, leaf, fruit) of control and 3HFWC nanosubstance grown cherry tomato plants were used for histological analysis. The preparation of steam tissues was assessed by transversal or longitudinal sections of plant material by hand, using a razor blade. Thin samples of steam sections, fresh or bleached leaves (acetic acid/glycerol/ethanol (1:1:3; *v*/*v/v*) solution for 5 min at 100 °C) and fruit parts were placed on microscopic slides in a drop of glycerol and covered with cover slips before microscope analysis. All samples were analyzed by a light microscope (Nikon Eclipse E100) and photographed by MicroCam SP 5.1 Bresser.

### 4.4. Photosynthetic Pigments Content

Photosynthetic pigments content (*Chl* and carotenoids) was evaluated spectrophotometrically according to [83]. Pigment content was measured in leaves of control and 3HFWC nanosubstance-grown plants after 4 and 12 weeks of growth in GrowBoxes. Ethanol extracts of frozen leaf samples (0.02 g) were incubated in a water bath at 70 °C for 10 min and cooled in the darkness. The absorbance of extract was measured at 470, 648, and 664 nm with a UV-visible spectrophotometer (Agilent 8453, Santa Clara, CA, USA) The concentration of photosynthetic pigments is presented as mg g^−1^ FW.

### 4.5. Oxidative Stress Assessment

#### 4.5.1. Histochemical Localization of O_2_^−^ and H_2_O_2_ Production

Superoxide anion (O_2_^−^) and H_2_O_2_ accumulation were detected in the leaves of control and 3HFWC nanosubstance-grown plants after 12 weeks. Localization of O_2_^−^ was evaluated using the nitro blue tetrazolium (NBT, Sigma-Aldrich, St. Louis, MO, USA), while H_2_O_2_ accumulation was evaluated using the 3,3′-diaminobenzidine (DAB, Sigma-Aldrich, St. Louis, MO, USA) staining method [84]. Leaves of control and 3HFWC nanosubstance-grown plants were excised and immersed for 2 h in 0.2% solution of NBT prepared in 50 mM sodium phosphate buffer (pH 7.5) and 1.25 mg/mL DAB-HCl (pH 3.8). The samples were wrapped in aluminum foil and incubated at room temperature. The localization of endogenous O_2_^−^ in leaf tissue was imaged as blue formazan precipitates as a result of the reduction of pale yellow NBT by O_2_^−^. At the sites of endogenous H_2_O_2_ accumulation in leaf tissue DAB forms a deep brown polymerization product in the presence of the peroxidases [85]. As a staining protocol control, some leaf samples were incubated in buffer or 10 mM ascorbic acid. *Chl* from leaf tissue was extracted with acetic acid/glycerol/ethanol (1:1:3; *v*/*v/v*) solution for 5 min at 100 °C or until leaf color removed. The bleached explants were submerged in glycerol/ethanol (1:4; *v*/*v*) storing solution before mounting on slides for a light microscope (Nikon Eclipse E100) observation. The pictures were taken using a BresserMicroCam SP5.1 device camera.

#### 4.5.2. MDA and H_2_O_2_ Content Analysis

MDA, product of lipid peroxidation, was measured according to the method by [86] using the 2-thiobarbituric acid (TBA) reaction. Leaf tissues (0.10 g) were homogenized in the liquid nitrogen with 0.1% trichloroacetic acid (TCA) and centrifuged at 15,000× *g* at 4 °C for 10 min. After that, mixture of 20% TCA and 0.5% TBA was added to the supernatant and incubated in water bath at 95 °C for 30 min. The samples were immediately placed on ice to stop the reaction. The mixtures were centrifuged at 15,000× *g* at 4 °C for 5 min and absorbance of the red adduct was recorded at 532 and 600 nm using MicroPlate Reader (LKB 5060-006, Vinooski, VT, USA). For the quantitative detection of H_2_O_2_ the extract was prepared in the same way as for MDA and centrifuged at 15,000× *g* at 4 °C for 15 min. The supernatant was added to the reaction solution containing 10 mM potassium phosphate buffer (pH 7.0) and 1M KJ. The mixture was used for absorbance measurement with a microplate reader at 390 nm [87].

#### 4.5.3. Antioxidant Enzyme Activity

Frozen foliar tissue (1 g) of control and 3HFWC nanosubstance-grown plants was grinded in liquid nitrogen and homogenized with chilled extraction buffer (50 mM Tris pH 8, 1 mM EDTA, 30% glycerol, 1.5% poly-vinylpolypyrrolidone (PVPP), 10 mM dithiothreitol (DTT) and 1 mM phenyl-methylsulfonylfluoride (PMSF). The homogenates were centrifuged at 12,000× *g* at 4 °C for 10 min. The obtained supernatant was used for determination of protein concentration with the Bradford method [88], and after that, the antioxidant enzyme activity assays. The activities of superoxide dismutase (SOD), catalase (CAT), peroxidase (POX), were determined according to the protocols described by [89,90,91]. Measurement of SOD activity was based on the principle that one unit of SOD activity is defined as the amount of enzyme that would inhibit 50% of NBT photo reduction to blue formazan at 560 nm. The reaction mixture for SOD included 50 mM phosphate buffer (pH 7.6), 13 mM methionine, 750 mM p-nitro blue tetrazolium chloride (NBT), 4 μM riboflavin and 0.1 mM EDTA mixed with the protein extract. CAT activity was evaluated by monitoring the absorbance decrease of H_2_O_2_ for 1 min at 240 nm (extinction coefficient of 0.0436 mM cm^−1^). The reaction mixture for CAT (3 mL) included: 50 mM Na-K-phosphate buffer (pH 7) and 20 mM H_2_O_2_. The reaction was initiated by adding 20 μL of protein extract in reaction mixture. POX activity was assayed using H_2_O_2_ and pyrogallol (Sigma-Aldrich, St. Louis, MO, USA) as the reaction substrates at 470 nm (extinction coefficient 2.47 mM cm^−1^). The reaction mixture for POX (3 mL) included: 50 mM K-phosphate buffer (pH 6.5), 20 mM pyrogallol, 10 mM H_2_O_2_ and 10 µL of crude enzyme. All the enzymes’ activities were expressed as μmol min^−1^mg^−1^ of soluble protein (U mg^−1^).

#### 4.5.4. Total Polyphenol Content

Total polyphenols were extracted following method described in [92]. Polyphenols from plant extracts react with Folin–Ciocalteu reagent (FC) and due to formation of blue-colored complex, quantification can be evaluated spectrophotometrically. Briefly, the reaction mixture contained 300 μL of FC reagents solution (FC/water; 1:2), 1340 μL deionized water and 60 μL of supernatant of ethanol leaf extracts. After quick vortex and left at room temperature (5 min) in 300 μL pf 20%, Na_2_CO_3_ was added and left for 90 min in dark conditions. Absorbance of reaction mixture was measured at 765 nm with gallic acid as a standard for phenol. Total polyphenol content was calculated as mmol of gallic acid equivalents/g extract.

#### 4.5.5. DPPH Radical Scavenging Capacity Assay

The free radical scavenging capacity of control and 3HFWC nanosubstance grown cherry tomato was measured using 2,2′-diphenyl-1-picrylhydrazyl (DPPH) assay according to the method described earlier [93,94]. DPPH is commercially available violet colored stable radical which in reaction with antioxidants from plant extracts convert to non-radical pale yellow colored form. The stock solution was prepared by dissolving 4.5 mg DPPH with 45 mL methanol, while the working solution has adjusted by diluting with methanol, so the absorbance of prepared DPPH solution was below 1.0 at 520 nm. The reaction mixture (400 μL DPPH, 500 μL methanol and 100 μL supernatant of leaf extracts) was incubated for 60 min in darkness at room temperature. The control simples were prepared as described above, with methanol instead of the plant extract. The scavenging activity of plant extract (%) was estimated based on the DPPH radical scavenged as the following equation: Scavenging capacity of plant extract (%) = [1 − (absorbance of plant extract sample − absorbance of control sample)/(absorbance of control sample)] × 100.

### 4.6. Aquaporin Genes Expression Analysis

Aquaporin genes expression analysis was performed in leaf samples of 3HFWC nanosubstance and control plants after long-term growth in the GrowBoxes.

#### 4.6.1. RNA Isolation and Reverse Transcription PCR (RT-PCR)

Total RNA was isolated from leaves (100 mg) according to the method of [95]. RNA was quantified by using a NanoDrop spectrophotometer (NanoPhotometer^®^ N60, IMPLEN, Munich, Germany), and its quality and integrity were estimated by electrophoretic separation on 1.5% agarose gel. The samples were treated with DNase I (Thermo Fisher Scientific, Waltham, MA, USA) at 37 °C for 10 min to eliminate traces of DNA, according to the manufacturer’s protocol. The cDNAs were synthesized in the reverse transcription reaction (RT) from 1 µg of total RNA. The reaction mixture for RT, in volume of 21 µL, contained 10 µL of total RNA (0.1 µg/µL), 25 mM MgCl_2_, 1 mM dNTP, inhibitor RNA-asa (20 U/µL), random hexamers (50 µM) and 15 U of MultiScribe^®^ transcriptase.

#### 4.6.2. Quantitative Real-Time PCR (qRT-PCR)

Relative expression of aquaporin genes was measured by quantitative real-time RT-PCR (qRT-PCR) using SYBR green in QuantStudio 3 Real-Time PCR System (Applied Biosystems, Foster City, CA, USA). Expression levels of *SlPIP1;3* (GenBank™ accession number: AB845606), *SlPIP1;5* (GenBank™ accession number: AB845607) and *SlPI2;4* (GenBank™ accession number: NM_001247696.2) were measured by qRT-PCR method in 10 μL qPCR reaction containing 1 μL of RT reaction product, appropriate forward and reverse primers and Maxima SYBR Green/Rox qPCR Master Mix (Thermo Fisher Scientific, Waltham, MA, USA). Primer sequences for *SlPIP1;3* and *SlPIP1;5* were obtained from the work of [96], while the primer for *SlPIP2;4* was obtained from the work [97]. Thermal cycling conditions for qRT-PCR included: initial denaturation on 95 °C, then 40 cycles of denaturation (95 °C for 30 s), annealing (57 °C for 30 s for *SlPIP1;3* and *SlPIP1;5*, and 70 °C for *SlPIP2;4*) and extension (72 °C for 30 s). For each sample, qRT-PCR was performed in triplicate. The expression levels of tested aquaporin genes were normalized to the universal housekeeping gene 18 s RNA and calculated relative to control from the greenhouse, according to the ΔΔCt method [98]. For each gene, one-way ANOVA was used for normality evaluation followed by Duncan’s multiple range tests (*p* ≤ 0.05) to estimate the significance of ΔΔCt differences between control and 3HFWC+ nanosubstance-treated plants.

### 4.7. Determination of Lycopene Content and BRIX

The content of lycopene in lipophilic extracts was determined by the HPLC-DAD method. Hexane extract (10 mg) was dissolved in 10 mL of mobile phase B (acetone-methanol 75:25, *v*/*v*). The solutions were filtered through a 0.45 μm regenerated cellulose membrane (Agilent, Paolo Alto, CA, USA) before injecting into the HPLC system. The HPLC analysis was performed on a liquid chromatograph (Agilent Infinity 1260 series, Paolo Alto, CA, USA), equipped with a diode array detector (DAD), on a Zorbax^®^ C18 column, 3 μm, 3 mm × 250 mm. Acetone: water mixture (75:25, *v*/*v*) was used as mobile phase A. The gradient used at a flow rate of 1500 mL/min was 50% A and 50% B at baseline; 0% A, 100% B after 15 min; 50% A, 50% B after 20 min. The analysis time was 20 min, and the subsequent time was 5 min. A 10 µL sample was injected into the system using an auto sampler. The spectrum was recorded in the range of 250–600 nm, and the chromatograms at 460 nm. Lycopene was identified in the samples according to retention times and spectral characteristics in comparison to standards, while the external standard method was used for the quantification. The 1 mg/mL lycopene stock solutions were prepared by dissolving the accurately weighed mass of commercial standards in mobile phase B. The solutions used to construct the calibration curve were prepared by diluting the stock solutions. The linear regression equation, obtained on the basis of the dependence of the area under the peaks on the chromatogram on the known concentrations of the standard, was used to calculate the concentration of individual carotenoids in the samples. The determinations were carried out in triplicate.

### 4.8. Statistical Analysis

All experiments were performed in triplicate, and each experimental group consisted of 12 plants. The results were expressed as the mean ± standard error, and statistical analyses were performed using the StatGrafics software version 4.2 (STSC Inc., Rockville, MD, USA). For the analysis of ANOVA variance, the LSD test with a significance level of *p* < 0.05 was used.

## 5. Conclusions

According to results obtained in this study, the 3HFWC nanosubstance exposed to the cherry tomato plants showed beneficial effects on seed germination and overall plant growth. Additionally, exposure to the 3HFWC nanosubstance improved tomato antioxidant defenses, decreased ROS production, and improved aquaporin gene expression. Most significantly, cherry tomato fruits accumulated more lycopene after exposure to the 3HFWC nanosubstance. Considering all the obtained results, it can be concluded that 3HFWC nanosubstance is safe to use, has no adverse effects on cherry tomato growth, and might be considered as a possible candidate for use as a nanofertilizer.

## Figures and Tables

**Table 1:** The effect of carbon nanoparticles on tomato growth and development.

Type	Concentration	Application	Effect	References
Nanotubes (single walled) SWCN	104, 315, 1750 mg/L	In vitro	Root elongation inhibition	[23]
	50 μg/mL	In vitro	Biomass increase	[24]
	50 μg/mL	In vitro	Biomass increase	[25]
	0–40 mg/L	In vitro/ex vitro	Seed germination and growth improved in vitro/shoot growth retardation ex vitro	[26]
	1 or 10 mg/kg	Soil	Delay of growth and flowering	[37]
SWCD-QD	50 μg/mL each	In vitro	Increased leaf senescence, root growth inhibition	[25]
SWCN-OH	1 or 10 mg/kg	Soil	Delay of growth and flowering	[37]
SWCNT-COOH	1 mg/10 mL	Overnight dip	Increased biomass production	[27]
Nanotubes (multi walled) MWCN	50 μg/mL	In vitro	Fresh biomass increase,	[47]
	40 μg/L	In vitro	Increased germination rate and biomass	[28]
	500–5000 mg/kg	Vermiculite	Unaffected to fresh biomass	[38]
	50–200 μg/L	Soil	Significant increase in plant height, flower and fruit formation	[39]
	50 mg/L (0–60 min)	In vitro	Seed germination and seedlings length increased	[29]
	50 mg/L	Hydroponic system	Increased fruit weight and number	[34]
	1 or 10 g/kg	Soil	Delay of growth and flowering	[37]
	100 mg/L	Foliar application	Increasing fruit yield& decreasing pathogen	[33]
Nanohorns	25, 50, 100 μg/mL	In vitro	Increased seed germination	[30]
Graphene	500–2000 mg/L	Hydroponic culture	Inhibited plant growth and biomass	[35]
	40 μg/L	Seed pretreatment	Better seed germination, longer steam but less biomass	[31]
Graphene oxide	20, 50 mg/L	In vitro	Improve biomass and root system	[32]
Graphene-QD	0–1500 mg/L	In vitro/hydroponic	Growth inhibition on higher concentrations	[36]
Fullerene	40 mg/L	Vermiculite	Unaffected on fresh biomass	[40]
	500–5000 mg/kg	Vermiculite	Unaffected to fresh biomass and pesticide accumulation	[39]
Fullerol C_60_(OH)_20_	50 mg/L (0–60 min)	In vitro	No effect on seed germination and plant growth	[29]

**Table 2:** Growth characteristics of control and 3HFWC nanosubstance grown cherry tomato seedlings.

Treatment	Seedlings		
	Height (cm)	Weight (g)	Average Leaf Number
Control	7.37 ± 0.13 ^a^ *	0.24 ± 0.08 ^a^	5.00 ± 0.10 ^a^
3HFWC	12.65 ± 0.81 ^b^	0.83 ± 0.03 ^b^	5.70 ± 0.33 ^b^

* The data represented in table are mean value ± standard error. Mean values within the same column marked with different letters are statistically different according to Fisher’s LSD test (*p* ≤ 0.05).

**Table 3:** The effect of 3HFWC nanosubstance on the morphological characteristics of tomato fruits.

Plants	Fruit		
	Weight (g)	Width (mm)	Length (mm)
Control	6.85 ± 0.20 ^a^ *	12.13 ± 0.10 ^a^	19.41 ± 0.36 ^a^
3HFWC	9.07 ± 0.30 ^b^	13.20 ± 0.33 ^b^	21.91 ± 0.24 ^b^

* The data represented in table are mean value ± standard error. Mean values within the same column marked with different letters are statistically different according to Fisher’s LSD test (*p* ≤ 0.05).

## Data Availability

Not applicable.
